# Thermographic Microstructure Monitoring in Electron Beam Additive Manufacturing

**DOI:** 10.1038/srep43554

**Published:** 2017-03-03

**Authors:** J. Raplee, A. Plotkowski, M. M. Kirka, R. Dinwiddie, A. Okello, R. R. Dehoff, S. S. Babu

**Affiliations:** 1 Department of Mechanical, Aerospace, and Biomedical Engineering, University of Tennessee, Knoxville, Tennessee, USA; 2 Manufacturing Demonstration Facility, Oak Ridge National Laboratory, Knoxville, TN, USA; 3 Materials Science & Technology Division, Oak Ridge National Laboratory, Oak Ridge, TN, USA

## Abstract

To reduce the uncertainty of build performance in metal additive manufacturing, robust process monitoring systems that can detect imperfections and improve repeatability are desired. One of the most promising methods for *in situ* monitoring is thermographic imaging. However, there is a challenge in using this technology due to the difference in surface emittance between the metal powder and solidified part being observed that affects the accuracy of the temperature data collected. The purpose of the present study was to develop a method for properly calibrating temperature profiles from thermographic data to account for this emittance change and to determine important characteristics of the build through additional processing. The thermographic data was analyzed to identify the transition of material from metal powder to a solid as-printed part. A corrected temperature profile was then assembled for each point using calibrations for these surface conditions. Using this data, the thermal gradient and solid-liquid interface velocity were approximated and correlated to experimentally observed microstructural variation within the part. This work shows that by using a method of process monitoring, repeatability of a build could be monitored specifically in relation to microstructure control.

Metal additive manufacturing (AM) is a process for fabricating solid metal parts through layer by layer build-up of metal in the form of powder, wire, or thin sheets^1^. Metal AM provides numerous benefits over traditional methods of production such as providing high geometric flexibility, decreased lead times, reduced energy consumption, and reduced waste^2^. Extensive research is being performed to further develop the technology and meet industry needs so that widespread adoption of the technology can take place. One method of metal additive manufacturing is electron beam melting (EBM), which is a powder bed fusion process that melts sintered powder using electrons generated by a heated filament^1^. In this process, an EBM machine first uses a re-coater blade to “rake” a thin layer of powder across the powder bed within a vacuum chamber. An initial scan of the electron beam preheats and lightly sinters the powder, which is then selectively melted according to the cross-sectional geometry of the current layer, and then reheated to normalize the temperature of the powder bed before repeating for the next layer. EBM typically produces less residual stress build-up during the build and is faster than other methods of powder bed fusion due to its high preheat temperature and use of a high speed electron beam, respectively^1^. Electron beam AM has also shown the ability to control local microstructure, depending on the process parameters used during the build process^3^.

Although additive manufacturing can provide numerous benefits for rapid prototyping and building complex geometries, there are limitations in surface finish, achieving desired tolerances, and process qualification and repeatability^2^. EBM specifically faces issues with *in situ* aging of the material’s microstructure due to prolonged exposure to elevated temperatures during processing, overheating of the material which can lead to issues such as swelling and cracking, and a less favorable surface finish due to its use of a relatively large layer thickness and coarser powder^1^,^4^,^5^.

In an attempt to better understand the AM process and reduce the need for costly experimentations, several models of the melt process have been developed at various length and time scales (microstructural, particle interaction, macroscale thermomechanical responses, etc.)^1^. However, if these models are to be more widely implemented, there is a need for high quality validation data in order to establish the accuracy of these model predictions. By connecting these models with actual process data they can be validated or modified to better predict the outcome of a process and improve reliability.

For these reasons, many recent research efforts in additive manufacturing have focused on utilizing *in situ* monitoring to further understand AM processes and to develop methods for real time process control^6^,^7^,^8^,^9^. This process control could provide a robust way for AM machines to alter process parameters in real time and adjust for the various factors that contribute to a build’s success or failure. Currently, the main methods of process monitoring for EBM are thermographic imaging, pyrometry, and thermocouples within the substrate^1^. However due to the nature of pyrometers and the location of the thermocouples, the most useful method currently in use for thermal process monitoring of the build is thermographic imaging with infrared cameras^1^,^10^.

Thermographic imaging is a non-contact method to optically monitor the temperature of an object using an infrared (IR) camera^11^. An IR camera utilizes a sensor to collect the infrared radiation that is focused through a lens and converts this information into a digital image. This information can then be converted into a temperature profile by assigning a temperature to the relative IR intensity of the region. However, the IR intensity observed by the camera is directly dependent on the emittance of the material surface, which is a function of both the material emissivity (an intrinsic material property defined for optically smooth surfaces) and the surface conditions requiring calibration for specific situations^11^,^12^. This dependence of emittance on surface conditions becomes an issue when monitoring the EBM process due to the changes in the surface topology as a part melts from powder into a solid.

Emissivity is the ratio of the radiation emitted by an optically smooth surface to the radiation emitted by a blackbody at the same temperature^13^. Due to an IR camera’s reliance on the emittance of a material, issues arise when trying to monitor an object that undergoes dramatic changes in emittance, such as during the EBM process. The material’s emittance changes during the EBM process because, as the part changes from sintered powder to a melted as-printed surface, the surface topology moves from a porous surface covered in highly emissive holes to a flat surface with lower overall emittance. This drop in emittance has been modeled and experimentally confirmed by Sih and Barlow using equations (1,2,3) for the emittance of holes in a porous powder bed and comparing them to an as-printed surface^14^


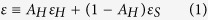



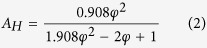



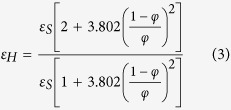


where *ε, ε*_S_, and *ε*_*H*_ are the overall observed emittance, the emittance of the printed surface, and the emittance of pores in the powder respectively, *A*_*H*_ is the fractional area of the surface that is occupied by porous holes, and *φ* is the fraction space in the powder volume that is unoccupied. The constants in the function come from assumptions based on the amount of pores that occur in between particles in the powder bed. The emittance of Inconel 718 has also been determined to exhibit this change in surface emittance with experimental measurements of the powder having an emittance of 0.68 and the as-printed surface exhibiting a range of emittance that is linearly dependent on the temperature of the material from 0.37 at a temperature of 800 °C to 0.46 when the material is observed at 1275 °C^12^. This change in emittance proves to be a problem for monitoring of AM processes because it introduces significant error when converting IR intensity to usable temperature data if it is not properly accounted for. This inaccuracy is seen when thermographic imaging of a part undergoes an apparent drop in intensity as the powder is heated above its freezing range and changes from sintered powder to a solid metal part (Fig. 1).

This study presents a method for processing IR data collected for EBM to accurately represent the transient layer temperature for both the powder and as-printed surface conditions. The properly calibrated temperature data is then used to quantitatively estimate thermal parameters that affect the solidification structure of the final part. The specific parameters of interest are the thermal gradient of the material, G, and the solidification interface velocity, R. It is well known from the solidification and welding literature that these parameters play an important role in the competition between equiaxed and columnar grain growth^15^,^16^,^17^. Under standard conditions, EBM components generally exhibit columnar microstructures, but by application of these principles to additive technologies, Dehoff *et al*. were able to obtain site specific control of grain texture in IN718 based on the scan strategy used^3^,^18^,^19^. The goal of this work is therefore to use IR thermography to demonstrate the monitoring of grain structure development within an additively manufactured component in order to enable future control systems designed to reliably produce on-demand microstructure control.

## Methodology

### Part Geometry and Design

**Figure 2:**
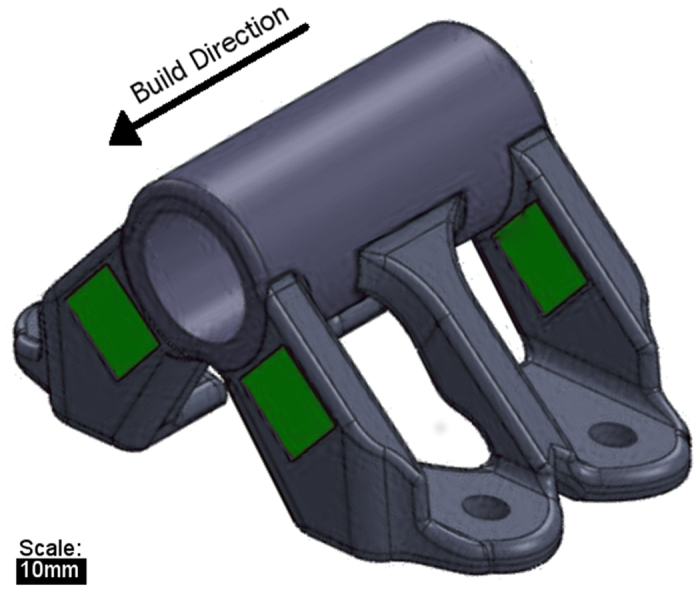
The part geometry used in the study. The regions highlighted in green are where a point melt strategy was used to melt the material, while the rest of the object underwent a line melt strategy.

**Figure 3:**
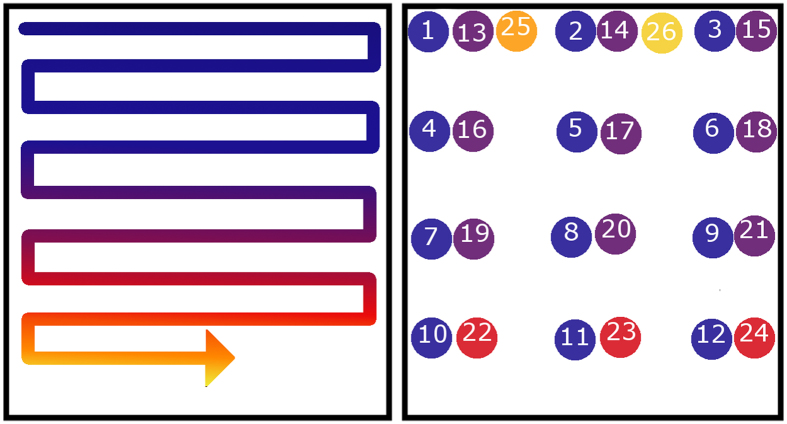
A graphical representation of the Line melt (left) and Point Melt (right) scan strategies.

The part built for this study was an IN718 bracket with four mounting holes produced in an ARCAM S12 EBM system (Fig. 2). The part has a constantly changing cross sectional geometry to encompass numerous different shapes as well as regions with two differing melt strategies (Figs 2 and 3). The design was created to utilize electron beam melting to control part microstructure and to test the effects of load strains on different microstructures that both occur within the same component. For the bulk of the build, a standard line scan melt strategy was implemented, in which the beam follows a path that moves side to side as it moves in one direction along the build (Fig. 3). As reported in the literature, this type of scan strategy produces a columnar microstructure with grains growing epitaxially from previous layers in both EBM and selective laser melting (SLM)^3^,^20^,^21^,^22^. In the two square regions (indicated in green in Fig. 2), a point melt scan strategy was used in which the electron beam was fired in a rapid series of single point melts that are offset by a certain distance from each other before the beam makes its return to start melting the next series of point melts in the pattern (Fig. 3)^3^,^23^. Using a pattern such as this has been proven to create an equiaxed grain texture that provides relatively isotropic properties as compared to the columnar texture produced by line scans which is generally strongest in the build direction due to the removal of grain boundaries that are transverse to the build direction^24^.

### Metallography

Following the build, selected regions of the as-built part were sectioned and prepared for metallography. The samples were ground and polished with standard techniques, then submersion etched in a mixture of HCl, acetic acid, and HNO_3_ (1:1:1). The microstructure was imaged with optical and scanning electron microscopy. To evaluate the crystallographic texture of the grain structure in various regions, electron backscatter diffraction (EBSD) was performed using a JEOL 6500 SEM.

### Camera Setup

In order to collect the thermographic data for this build a mid-wave IR camera—the FLIR 7600—was placed outside of the ARCAM vacuum chamber and aimed at the build plate through a leaded glass viewing window that was used to protect the camera’s sensor from radiation produced by the electron beam. Due to the space limitations inside of the ARCAM machine, the camera also had to be placed at an angle of 22 degrees to the build plate. To prevent metal vapor produced during the build from adhering to the viewing window, a roll of thin Kapton film was slowly scrolled in front of the viewing window to carry away the vapor as it collects on the film. By using this Kapton film to protect the window, it prevents any material build up from diminishing the accuracy of the IR images that were collected. The camera used a 25mm lens without a filter, the integration time was set at 86.4 μs, and the frame rate was set at 10 Hz. After the collection of all the thermographic imaging of the build, the data was then imported into FLIR’s proprietary imaging software ResearchIR for initial analysis and compilation of the data. Since the factory calibrations of the camera were not accurate for monitoring this build, the measurements collected were converted from the factory settings to the raw image intensity units, called counts in the software.

### Camera Calibration

**Figure 4:**
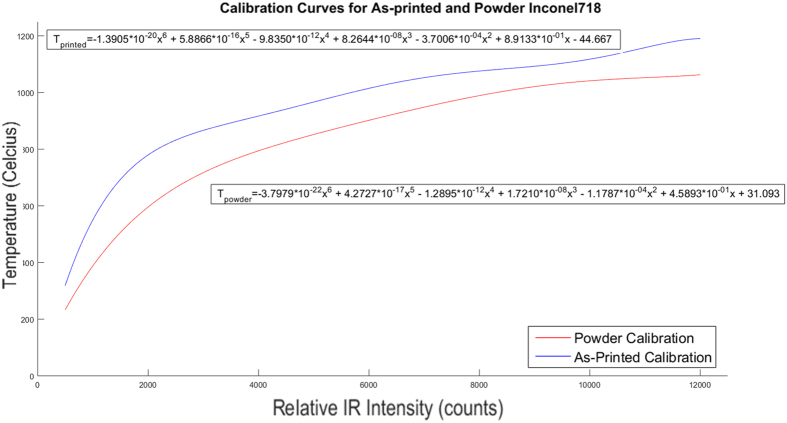
Intensity to Temperature calibration curves for Inconel 718 powder and as-printed parts shown with their respective equations. In the equations given T_powder_ refers to the temperature output gained from calibrating the intensity, denoted as x, to match the temperature that is representative of the powder emittance. T_printed_ is calibrated similarly but to match the temperature of the solid printed material.

**Figure 5:**
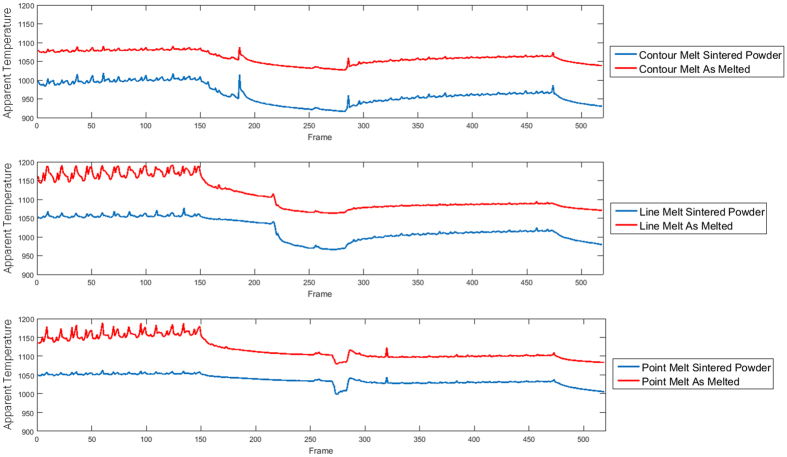
The Temperature Outputs from calibrations in FLIR’s ResearchIR software. Each chart created using FLIR’s software shows a different point in the build that underwent a differing scan strategy, the top shows the scan strategy for a point in the build that underwent the point melt strategy; the second, a contouring melt outlining the geometry of the layer; and the third shows a line melt strategy. The red line in each figure represents the calibration for the part as printed while the blue line shows the temperature of the point as calibrated for powder.

In order to gather more accurate thermal data, the thermal intensity of the part was calibrated for the specifics of this experimental setup, accounting for the particular camera and its view through the sheet of leaded glass which reduces the transmission of the radiation that the camera can detect. The same camera setup and calibration was used as outlined by Dinwiddie, *et al*. for Inconel 718^12^. In that work, calibrations for IN718 as a powder and as a solid metal were performed by plotting the infrared camera data (in counts) for each at known temperatures, measured using a type-K thermocouple and then finding the equation of best fit. The sixth degree polynomial equations determined using this method is shown for the sintered powder and as-printed part in Fig. 4 along with their respective equations. Using these calibrations, the temperature curve for the part for each of the emittance values at different points in the build layer can be converted from an intensity value to a temperature (Fig. 5).

### Processing Thermographic Data

For portions of the powder bed that are melted to produce the part geometry, obtaining accurate temperature histories depends on identifying the time at which the powder IR calibration should be substituted for the as-printed calibration, i.e. when melting occurs. In order to efficiently construct this composite temperature curve from the thermographic data, a way to automatically make this distinction using only the available IR data was developed.

**Figure 6:**
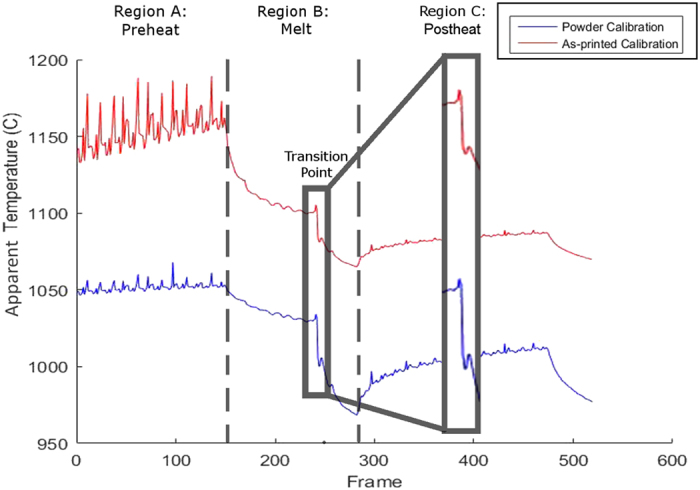
Temperature curve of the layer highlighting the EBM processes. Special attention needs to be paid to the transition point where the temperature rapidly drops due to the change in emittance. (Seen in the inlet).

As seen in Fig. 6, there are four distinct temporal regions of interest during the melt process for any point in the layer. In Region A, the part undergoes the initial preheat where there are rapid passes of the diffuse electron beam that results in cyclical heating and cooling that slowly brings the part up to temperature. Then, as the part enters Region B of the temperature curve, it begins to cool as the other regions of the build are melted. This cooling occurs until the beam reaches the pixel in question where there is a rapid drop off in apparent temperature due to the sudden change in emittance; this is the location of the transition point. Following this transition point, the pixel observed continues to cool as the remainder of the part is melted. Finally, the point begins to undergo the cyclical heating again as the post heat occurs until the end of the process where the layer begins to cool once more as the next layer of powder is raked across the build plate. In the collected thermographic data, the transition point occurs where there is a large drop in the apparent temperature of the part, almost 50 °C within 0.1–0.2 seconds for this case, but occasionally as much as 100 °C for other pixel locations.

**Figure 7:**
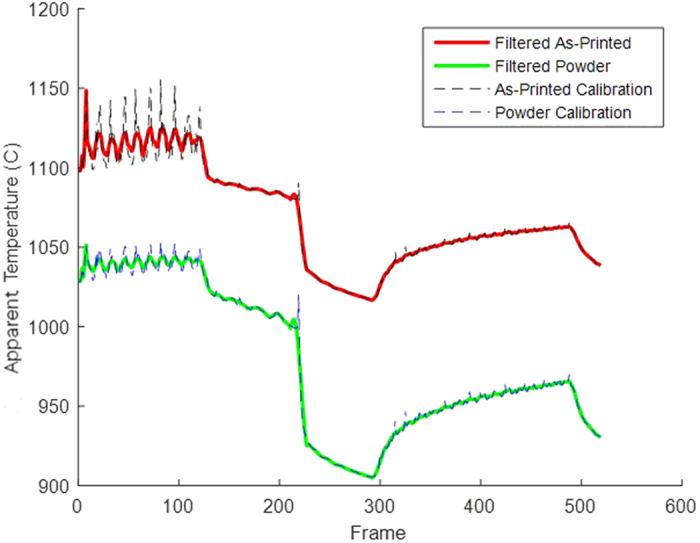
Filtered Temperature curve that removes some of the data’s noise. Filtering the data’s noise reduces the likelihood of selecting the transition from powder to solid part at the wrong frame.

The transition of the material from powder to solidified metal may be reliably identified by this drop in intensity during the melting region of the curve corresponding to a local minimum in the slope of the temperature curve. However, because the data has several regions with rapid intensity drops that do not correspond to melting, the data was smoothed using a simplified least squares method for each frame using the previous and subsequent eight frames (Fig. 7)^25^. By smoothing the data curve, the chances of selecting frames where rapid cooling occurred due to the actual temperature profile and signal noise, rather than a change in emittance was greatly reduced.

**Figure 8:**
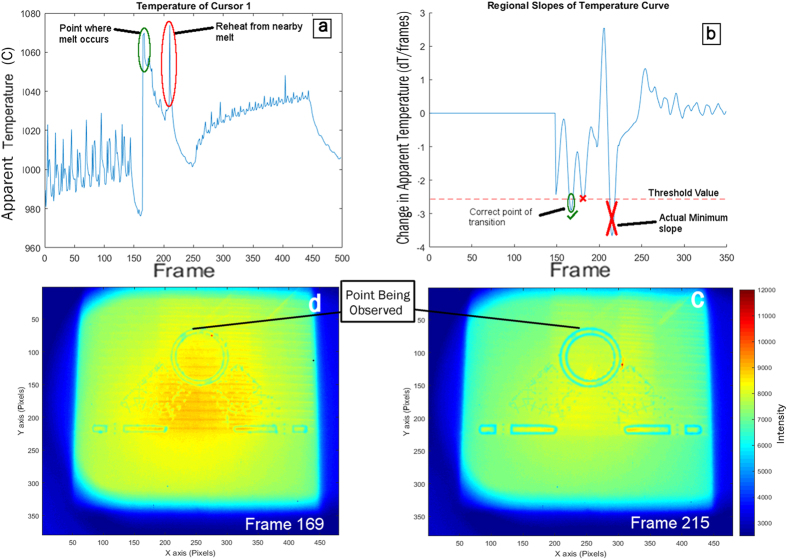
(**a**) Temperature curve showing the effects of reheating on the regional slopes created (**b**). If the absolute minimum slope was selected, the frame where reheating occurs (**c**) would have been selected rather than the true frame (**d**). When this particular contour point shown in the figure was being selected at frame 215 (**c**) rather than frame 169 (**d**) the part was beginning to melt the interior being filled by a line melt nearby which reheated the point causing the large temperature spike seen in (**a**) and the minimum slope value seen in (**b**).

Next, a regional slope value for each point in the filtered data was determined so that the location of the emittance change could be selected. To determine these regional slope values, the slope between the data points that occurred five frames prior to and five frames after the frame being observed was calculated and the slopes were recorded as a dataset over the entire length of the video before being processed further. Using a regional slope further prevented the algorithm from selecting a point where the instantaneous slope was large but not indicative of melting thus reducing the algorithm’s sensitivity to false-positives. With all of the regional slopes calculated, the frame with the minimum slope value was then selected as the melt point. However, the global minimum regional slope does not always correspond to the melting transition point. Occasionally, the point of interest is reheated by a neighboring scan of the electron beam, producing a temperature rise and fall that often caused a false positive to occur much later than the actual frame at which that point actually melted. In order to improve the selection, a threshold value for transition point detection was set as a percentage of the minimum slope, and the first frame with a slope that passed this threshold was selected. An explanation of this thresholding is shown in Fig. 8 where a point on the contour of the build is reheated by a line melt later in the process. In this build the detection threshold was set at 70% of the minimum slope.

**Figure 9:**
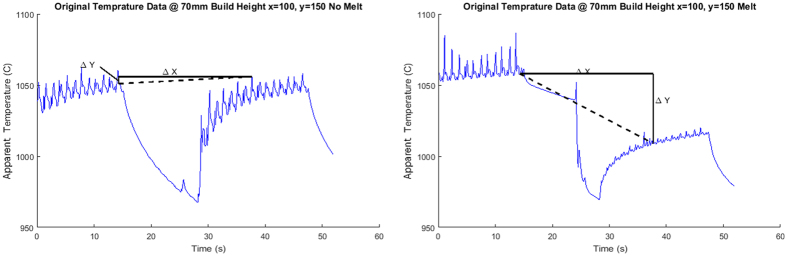
Comparison of the slope of the melting regions of a point in the build that did not melt (**a**) and one that did (**b**). Notice how the slope of the melted point is significantly lower than the no-melt region. The black lines in the figure show the relative change in frames and temperature of the point being observed. Notice the slope of the melting time frame on the left (**a**) is much shallower than the melting region of the curve on the right (**b**). Since the point on the left did not meet the threshold for detecting the melt the point in (**a**) did not melt.

However, one issue still remained: the algorithm must be able to automatically identify points that do not melt during the process. To determine whether or not melting occurred for a particular pixel (the melt condition), the region of the temperature curve where the melt occurs was analyzed for both points in the build that melted and those that did not. It was observed that when a point underwent melting the intensity drop that occurs due to the change in emittance of the material was far greater than the decrease in temperature that occurred as due to general cooling of the region. So, in order for the algorithm to detect the melting condition, the slope of the timeframe in which the melt occurred (Region B in Fig. 6) up to when the post heat (Region C) started was compared to a set threshold value (in this case −0.01 °C/s). If the slope of this region of the temperature curve was less than the threshold value, no change in IR calibration was applied (Fig. 9).

**Figure 10:**
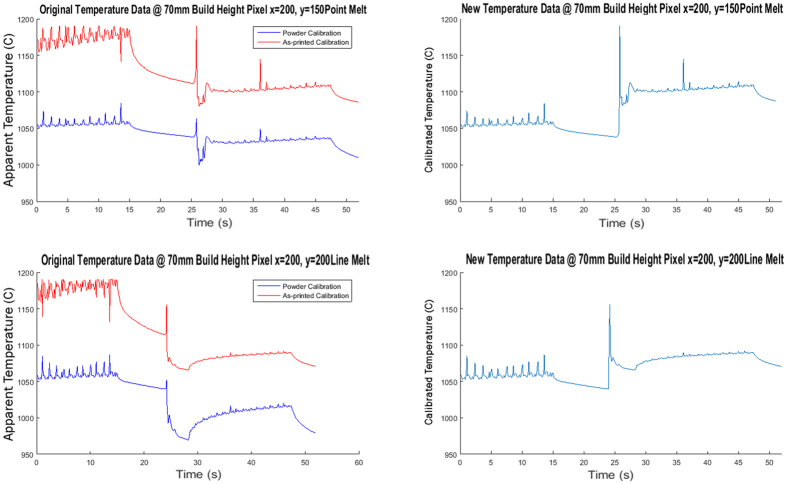
Composite temperature profile (Right) determined from the original calibration curve (Left) for two scan strategies (Point melt and line Melt).

Once a condition for melting was determined, the final step in the algorithm was to take the original temperature data for each camera calibration for IN718 (the sintered powder and the as-printed surface) and reconstruct a single temperature curve for each pixel. In order to do this, all temperature values before the transition were taken from the powder calibration and all of the points after the transition from the as-printed calibration. This final output provides a more accurate reflection of the transient temperature profile for any point in the build (Fig. 10).

**Figure 11:**
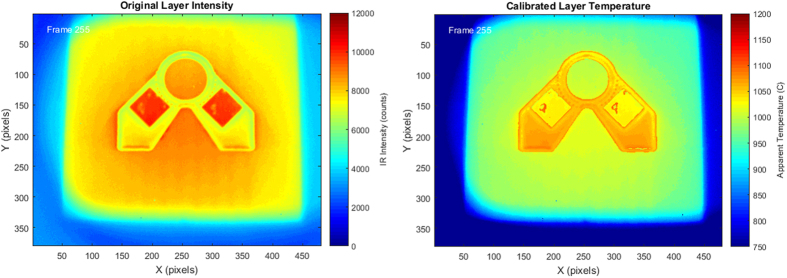
Comparison of final temperature output (right) to the original output (left).

**Figure 12:**
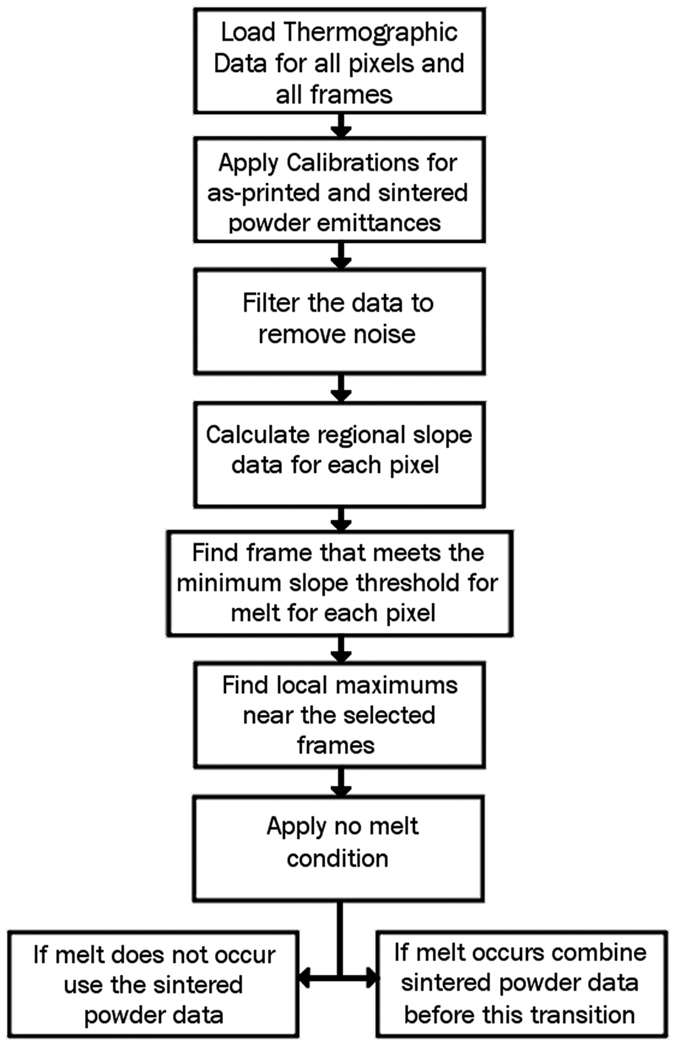
Flowchart of the algorithm process.

Once the algorithm was verified for a variety of individual pixels by comparison to the original thermographic output, the process was scaled up to handle large matrices of intensity values over an entire image for the full layer. These calculations were performed by changing all of the original vector functions into three dimensional matrix functions and performing bulk calculations to provide one large three dimensional matrix of temperature data for a layer during the melt process. This data was then plotted over time to produce the final video with the corrected temperature output (Fig. 11). By following the steps of this algorithm (Fig. 12) a more accurate temperature profile can be found and used for subsequent calculations and observation.

## Results

### Algorithm Output

Utilizing the above processes, the thermographic data collected during the build was processed and a final output was created for several layers within the build. Once processed, the data was able to give a much more accurate thermal profile of the build surface as the melt process occurs as seen in Fig. 11. The corrected temperature profile shows that the newly formed part is significantly hotter than the powder bed and the locations of the solidification front may be approximated. This corrected output shows that the algorithm is able to take the misleading raw output data from the IR camera and reconfigure it into a more useful set of quantitative temperature values for the surface as it melts during the build process.

In the final output of the thermographic data, several interesting phenomena were witnessed. Although the output as a whole more accurately portrayed the expected temperature profile, there were still select regions of the output that did not properly switch calibration curves which resulted in regions that appear cooler upon melting. Also, it was noted that the regions that underwent the point melt scan strategy appeared to be hotter than the line melt regions in the build. This temperature difference was maintained even after the part had ample time for all of the regions to come to an equilibrium temperature during the post heat procedure.

### Microstructure Observations

**Figure 13:**
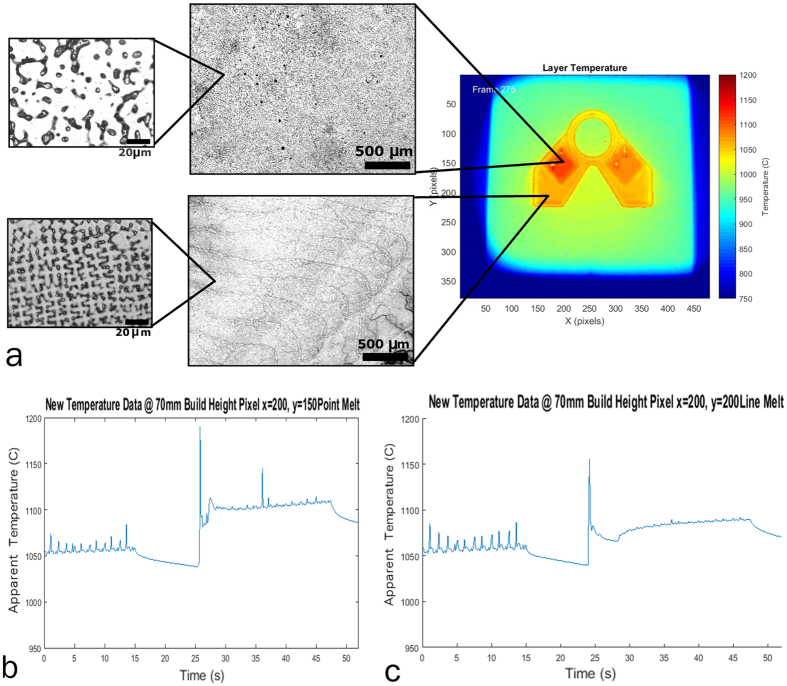
Relationship of the thermal history for the point melt and line melt (**b**,**c**) scan strategies and the microstructure of the material (**a**).

**Figure 14:**
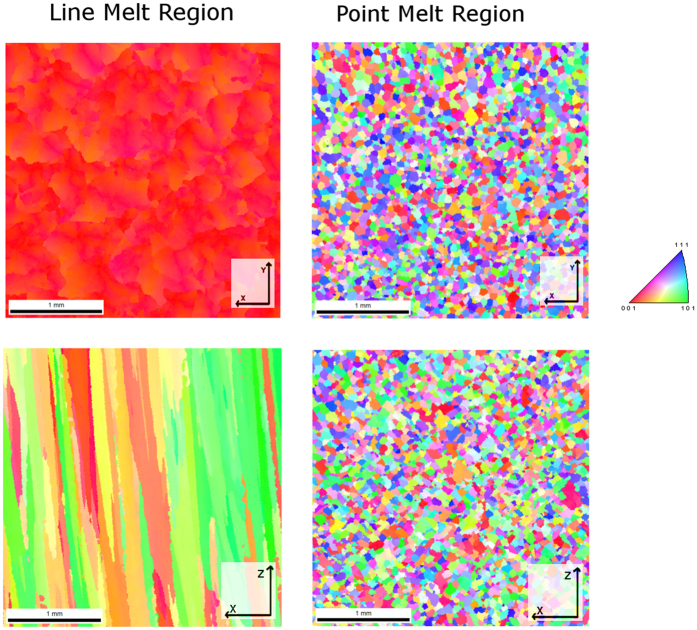
Results of the EBSD analysis. Each color in the images corresponds with the respective grain orientation designated in the legend on the right. The axes in the corner of each figure show the respective plane being observed within the material. The images on the left show long straight grains that grow epitaxially along the build direction (the z axis) whereas the right images show the randomly oriented grains of equiaxed grain structure.

Upon observing these micrographs of the material (Fig. 13), it can be seen that the point melt regions exhibit a coarser dendritic spacing than the line melt regions. This cross-section, taken in the x-y plane and normal to the build direction, shows that the line melt region contains highly oriented dendrites growing in the z-direction, while the growth direction in the point melt region is not easily identified. Electron Backscatter Diffraction (EBSD) (Fig. 14) confirmed that in the line melt regions, the part exhibits a columnar structure with the fast growth direction (<0 0 1>) oriented along the build axis (z-direction) and that in the point melt regions, the grains are randomly oriented. These microstructures are consistent with the observations made by Dehoff *et al*. for similar melt strategies in IN718^3^. Example thermal histories for each of these regions are shown in Fig. 13b,c.

## Discussion

### Correlations with Microstructure Development

Established theory for the competition between columnar and equiaxed grain growth shows that equiaxed microstructures are generally favored as thermal gradient decreases and the solid-liquid interface velocity increases^15^. This theory was used by Dehoff *et al*. to develop the melt strategies used here to control gain structure, and the effects of process parameters on the solidification conditions were further evaluated by Raghavan *et al*.^3^,^23^. Qualitatively, the difference in microstructure observed here may be rationalized by considering example thermal histories for each melt strategy (Fig. 13b,c). Following the melting of a pixel in the line melt region (Fig. 13c), the point cools off quickly while other regions are being melted, with some small increases in temperature due to neighboring line scans. In the point melt region however, following the initial temperature spike due to melting, the temperature rises due to local reheating of neighboring spots. These trends in the thermal histories are indicative of the thermal conditions during solidification, suggesting that the thermal gradient in the point melt region is lower than that in the line melt region, tending to favor equiaxed solidification.

Using the corrected temperature data gathered in this study, these differences in solidification conditions may be quantified. The thermal gradient at the part surface was calculated using a linear approximation of the temperature differences between pixels as shown in equations 4 and 5, where x and y are the corresponding pixel locations in the x and y direction of the image, and T_x_, T_y_, T_x+1_, T_x−1_, T_y−1_, and T_y+1_ are the temperatures of each of their corresponding subscripted pixel designations. These temperature differences were selected for the pixels just after the frame in which they melted in the build so that the thermal field most closely approximating the solidification conditions was used.


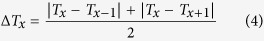



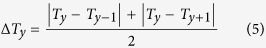


Based on a single still image of the build, ImageJ was used to determine a scale for the distance between the centroids of adjacent pixels in relation to known distances within the bracket. Since the camera is placed at an angle to the build, there is a small amount of distortion present that causes the pixel size to vary slightly as a function of location. These changes in pixel size were not accounted for in the thermal gradient and interface calculations, but this is a less significant source of error (~14%) compared to the inability of the present camera setup to resolve the beam size and location. The calculated average pixel size was 0.35 mm per pixel in the x-direction and 0.37 mm per pixel in the y-direction.

Using these distances and the temperature difference determined from equations 4 and 5, an estimate for the thermal gradient, G, was calculated using equation 6. Note that since the IR data only accounts for the surface of the build, the present analysis is limited to thermal gradients within the build (x-y) plane to approximate what is actually a three dimensional effect. Along with the thermal gradient, the solid-liquid interface velocity, R, was calculated using the temporal change in temperature that occurs immediately following the frame where the thermal gradient was calculated. The time derivative and the previously determined thermal gradient were used to calculate the solid-liquid interface velocity for every pixel in the thermographic image according to equation 7.


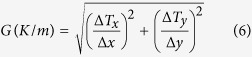



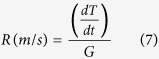


**Figure 15:**
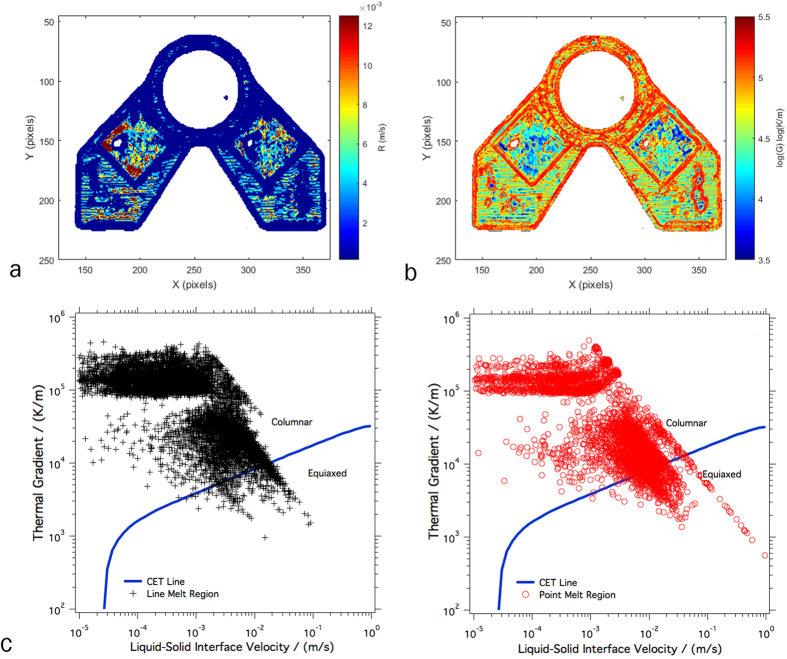
The layer Thermal Gradients (**a**), Interface Velocity (**b**), and CET curve (**c**). The interface velocity contour (**a**) shows that in the regions that are subject to the point melt strategy the velocity is relatively higher than the rest of the build. In the thermal gradient contour (**b**) the line melt regions tend to show gradients that are generally higher than the regions that underwent the point melt strategy. This follows with the literature and when all of the points are plotted on a columnar to equiaxed transition curve (**c**) there is a shift for the equiaxed region points toward the equiaxed region of the chart.

**Figure 16:**
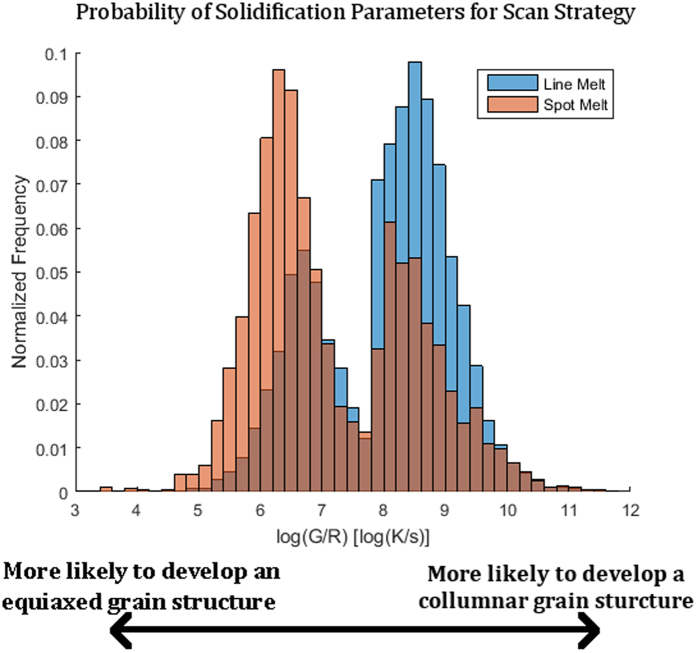
Histogram showing the distribution of the ratio of thermal gradient to the solid-liquid interface velocity. There is a clear shift for both toward the region where their respective grain structures would be more likely.

Figure 15a,b plot the approximated G and R values, respectively, for a particular layer over the geometry of the part. While there is significant noise in this data, it can be seen that, on average, the thermal gradient of the line scan strategy is generally higher than that of the point melt region and that the solid-liquid interface velocity of the line melt region is lower than the point melt region. When the distribution of G and R values for each melt strategy points are plotted and compared to an approximation of the columnar to equiaxed transition (CET) curve for IN718 (Fig. 15c), the pixels that were subject to the spot melt scan strategy move closer to the CET curve and begin to move into the region where a higher amount of equiaxed grains are present. Because increased thermal gradient favors columnar growth while increased solid-liquid interface velocity favors equiaxed growth, the difference between the two melt strategies and their effect on microstructure development may be best visualized by plotting the distribution of the ratio of these two parameters as shown in Fig. 16 as a histogram. While the distributions are wide, the data clearly indicates that points within the line melt region were significantly more likely to have a columnar grain structure whereas the point melt region strongly favors the development of equiaxed grain structures. These results based on the thermal data strongly correlate with the microstructures shown in Figs 13 and 14, suggesting that analyzing the IR data using the methodology proposed here may be a viable technique for monitoring, and eventual feedback control, of microstructure selection in EBM.

### Current Limitations and Areas for Further Development

Although the data shown in Figs 15 and 16 demonstrate the ability of the present technique to monitor the influence of thermal effects on the development of grain structure, significant noise in the measured data obscures the differences between melt strategies. While some of the sources of this noise are inherent to the technique (i.e. the IR camera is incapable of collecting subsurface data required to fully understand the three dimensional heat transfer effects), others may be mitigated in subsequent studies.

Perhaps the largest source of error is the time and length scales of the IR data collection relative to those at which the EBM process operates. The size of the pixels in this study was approximately 0.35 mm, which is too large to fully resolve the size of the melt pool. More significantly, the beam moves at a velocity as high as 5 m/s but the frame rate used here was only 10 Hz. For the given pixel size, this collection rate means that the beam may move a distance equivalent to more than 1000 pixels between images. Because the integration time (86.4 μs) is short compared to the time between frames, for most pixels, the actual time over which melting and solidification occurs was not captured. Given this limitation, it is encouraging that the present data was able to identify the difference in thermal conditions between columnar and equiaxed solidification. With increased camera resolution and frame rate, it is expected that this noise will be reduced significantly and the thermal gradients and interface velocities may be more accurately calculated, resulting in an improved distinction between solidification conditions. The tradeoff with increased resolution is the corresponding increase in data storage and post-processing computation requirements, so future work will require determination of the lowest temporal and spatial resolutions at which quality data may be obtained.

Another source of error is the lack of an IR calibration for the emissivity of the liquid metal. The origin of this limitation is in the calibration technique which relies on measurements from type-K thermocouples to monitor the temperature of the calibration target. The maximum temperature of the thermocouples is approximately 1200 °C, more than 100 °C below the liquidus temperature of IN718. While the calibration may be safely extrapolated to slightly higher temperature to account for the change in solid emissivity with temperature, no conclusions about the calibration for the liquid may be drawn. However, due to the limitations in spatial and temporal resolution of the IR data, only a very small fraction of data points would require this information. Therefore, this is an insignificant source of error for the present data set. If future studies use resolutions adequate to properly resolve the melt pool, an additional liquid calibration will be necessary.

In the final output of the model, the regions that utilized a point melt strategy maintained a much higher temperature value during the melt process even after the layer temperature was given sufficient time to equilibrate during the post heat. Upon examination of point melt regions that used the electron beam process, it was observed that the surfaces were characteristically rougher than the line melt regions. This surface roughness could be causing the emittance of the material to increase much like emissivity increases seen when holes in a material are observed. Recent research has confirmed the effect of build parameters on surface roughness and subsequent IR measurements in selective laser melting^26^. It is the goal of ongoing research to relate melt strategy to surface topology and IR intensity in EBM Similarly, powder size distribution and morphology is likely to have an effect on the calibration for the powder bed.

**Figure 17:**
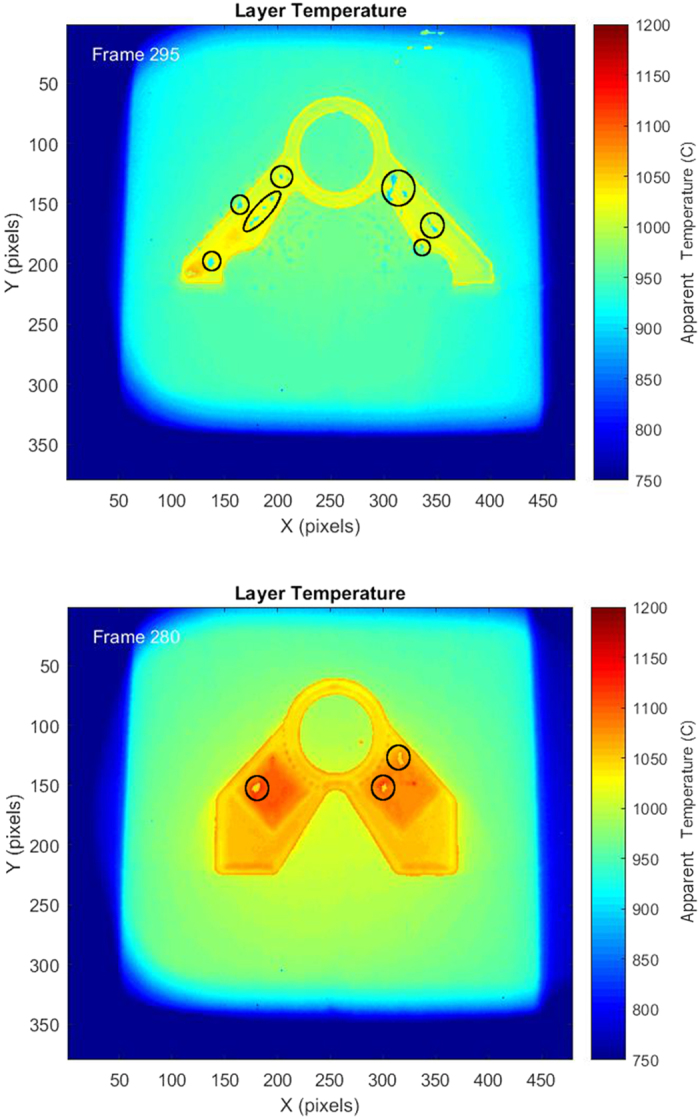
Layers with several algorithm faults where regions were marked as non-melting regions even though melting did occur at these regions.

**Figure 18:**
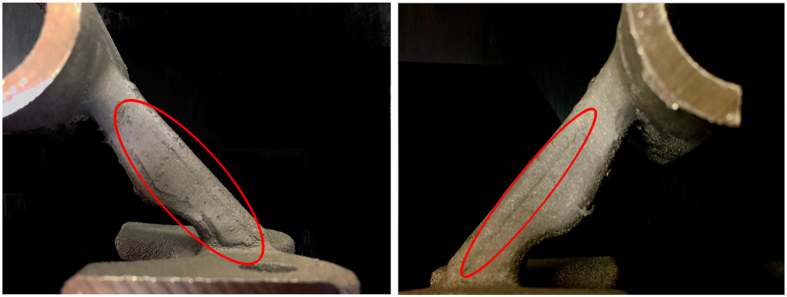
Images taken of the part after the build was completed. Surface swelling (right) and pitting (left) can clearly be seen in the part and could be a potential causes of algorithm failure.

**Figure 19:**
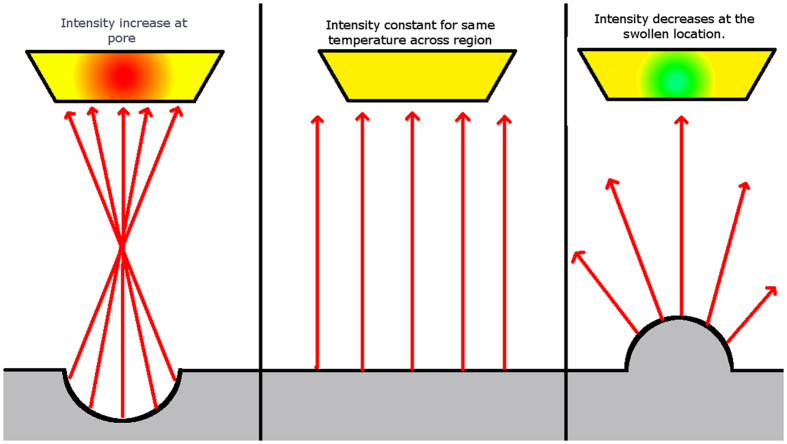
Comparison of relative intensity change as affected by pores (left) and swollen regions (right) to the original flat surface (center). Note that intensity increases for pores and decreases for swollen parts at the same temperature.

Other regions with abnormal IR data were also examined to determine what types of conditions would change the temperature data. In Fig. 17, there are regions that seem to vary from their surroundings even though the parameters for the melts there were the same as the surrounding material. Upon examination of the final part, it was observed that the material in this location had undergone swelling or pitting on the surface (Fig. 18). Because the swelling changes the geometry of the surface, the radiation from this region is directed away from the sensor of the camera and therefore reduces the observed IR intensity even though the temperature is similar to the surrounding material (Fig. 19). This has been quantified in Cheng *et al*., who showed that as an object is rotated away from an IR camera the intensity of the radiation is decreased^27^. This principle could provide a basis for surface swelling detection using *in situ* thermographic imaging.

**Figure 20:**
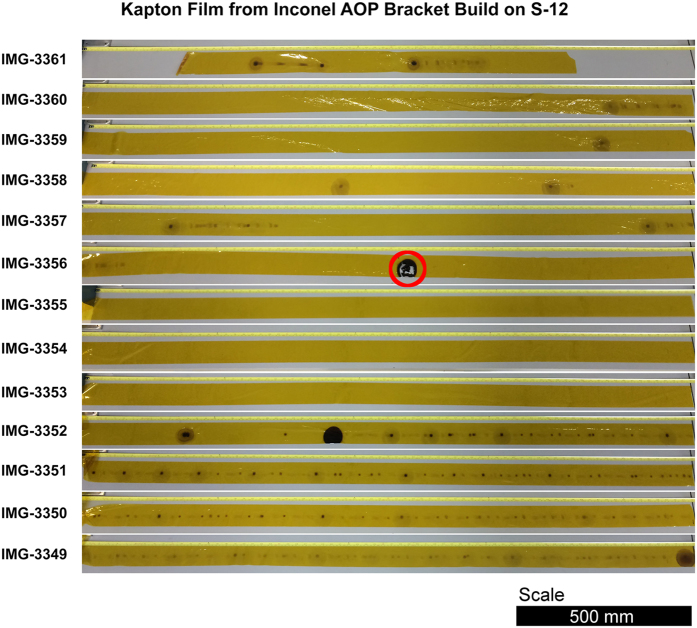
Image showing the metallization build-up on the Kapton film that protected the leaded window. The highlighted region shows where the film had melted during the build process.

Another area of concern was instances in which metallization occurred on the surface of the protective Kapton film used to keep the camera lens protected. Metallization is the result of condensation of metallic vapor created during the melting process onto the film as it is protecting the leaded glass window. In the images in Fig. 20 the film can be seen with large areas where metallization occurred. The operator noted that the film did jam at various times with one such occasion actually melting the film slightly. Metallization of the Kapton film changes the transmissivity of the material which reduces the amount of infrared radiation reaching the IR camera, resulting in a lower approximation of the calibrated temperature. Thus, the metallization of the film could have resulted in less accurate data.

Although this algorithm can provide a way to determine the surface temperature of an electron beam AM part, there are still issues that affect its ability to be used for real-time processing and feedback control. Due to its reliance on the entire temperature data set for a particular layer to determine the transition point and to determine the no melt condition, this method could not be used for true real-time processing. However, as it is currently designed, it can be used for intermittent feedback control of subsequent layers, meaning that, when certain phenomena are observed in a layer the next layer could be adjusted to avoid part defects and improve microstructure selection assuming that the cross-sectional geometry does not change dramatically. It could also be used to identify catastrophic failure to prevent machine damage from occurring. For instance, if it is observed that swelling is occurring within a part, the machine could alert the operator that they need to monitor the build more closely so that the part does not damage internal components such as the recoater blades. Also, this method could be used as a means to signal probable fault locations so that, when analyzed for quality control, these high-risk regions may be assessed more thoroughly for likelihood of failure. In order to determine a method for real-time monitoring and feedback control, further research in needed to validate and modify this model and determine a way to work around the need for an entire layer’s data to determine the transition.

There are opportunities to apply this methodology to other AM processes such as SLM or even polymer based fused deposition methods. Due to the difference in the thermal history of these processes, however, the material does not achieve as high of a temperature during the preheat. This change in temperature could affect the interpretation of the emissivity changes and the slope used to determine the time of the melt in the material. To use this methodology with other AM processes there is likely modification that will be required to be useful. However, once the difference in how the model operates during these processes is understood a similar method could be used to determine the true temperature profile in the part. Moving forward the process used in this study will be used for further research on these other processes.

## Conclusions

This study successfully developed a methodology for interpreting IR data in electron beam melting to account for differences in emittance between the powder bed and the as-printed component surface. The results show that by using a simple post-processing analysis of the IR intensity data, one can create a relatively accurate representation of the layer-wise temperature profile within an AM build. By properly transforming the IR intensity data into more useful temperature data in this way, more insight into the AM process was gained. The calibrated temperature data was used to approximate the thermal gradient and solid-liquid interface velocity in order to monitor the effect of thermal conditions on grain structure development. This methodology was able to distinguish between two different melt strategies, and based on existing microstructural development theory from the literature, correctly correlated the thermal data to regions of columnar and equiaxed grain growth. While these initial results are a promising proof of concept for the ability to eventually implement feedback control for both the thermal profile and microstructure distribution in EBM, additional research will be necessary to identify and mitigate sources of error in the IR measurements and to refine and reduce noise in the predictions of the thermal conditions.

Moving forward with this research, the present algorithm will be applied to several other builds specifically to understand the impact of process parameters and composition on IR calibration and the potential of using IR thermography to measure build repeatability. This processing will also be assessed for its viability for defect detection. Future work for this research will also aim to encompass other AM processes to expand the technology and its use.

## Additional Information

**How to cite this article:** Raplee, J. *et al*. Thermographic Microstructure Monitoring in Electron Beam Additive Manufacturing. *Sci. Rep.* **7**, 43554; doi: 10.1038/srep43554 (2017).

**Publisher's note:** Springer Nature remains neutral with regard to jurisdictional claims in published maps and institutional affiliations.

## Figures and Tables

**Figure 1:**
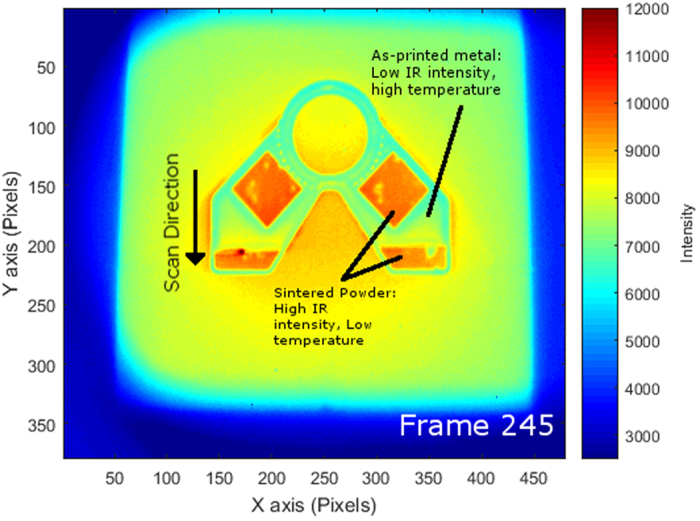
Original intensity data from the monitored build as interpreted by FLIR’s ResearchIR software. The scale on the right is the original output of relative intensity that is referred to as counts by the software. The image also shows the apparent drop in intensity as the electron beam scan moves across the surface of the build.
